# Evaluation of Long-COVID Syndrome in a Cohort of Patients with Endometriosis or Adenomyosis

**DOI:** 10.3390/jcm14061835

**Published:** 2025-03-08

**Authors:** Anjeza Xholli, Isabella Perugi, Francesca Cremonini, Ambrogio Pietro Londero, Angelo Cagnacci

**Affiliations:** 1 Academic Unit of Obstetrics and Gynaecology, IRCCS Ospedale Policlinico San Martino, 16132 Genova, Italy; anjeza.xholli@hsanmartino.it (A.X.);; 2 Department of Neurology, Rehabilitation, Opthalmology, Genetics, Maternal and Infant Health (DINOGMI), 16132 Genova, Italy; 3 Obstetrics and Gynecology Unit, IRCCS Istituto Giannina Gaslini, 16147 Genova, Italy

**Keywords:** endometriosis, adenomyosis, SARS-CoV-2 infection, long-COVID syndrome

## Abstract

**Background**: Long-COVID is characterized by the persistency of COVID-19 symptoms beyond 12 weeks, and it is probably consequent to immune dysregulation induced by SARS-CoV-2 infection. Immune dysregulation is associated with and probably involved in the pathogenesis of chronic gynecological conditions like endometriosis and adenomyosis. This study evaluated whether the presence of endometriosis or adenomyosis increases the risk of long-COVID, i.e., the persistence of COVID-19 symptoms beyond 12 weeks since infection. **Methods**: This retrospective observational study was performed at the outpatient service for endometriosis and chronic pelvic pain, at a university hospital. The diagnosis of endometriosis/adenomyosis was primarily based on clinical symptoms and ultrasonography assessment. Data regarding infection, vaccination, symptoms associated with SARS-CoV-2 infection, and their persistence for a minimum of 12 weeks were collected. **Results**: This study included 247 women, 149 controls without and 98 cases with endometriosis/adenomyosis. Among these, 194 (116 controls and 78 cases) had suffered from SARS-CoV-2 infection. Rates of infection and vaccination were similar in the two groups. The distribution of the SARS-CoV-2 vaccine was uniform across the two cohorts. COVID-19 patients with endometriosis or adenomyosis exhibited a higher prevalence (*p* < 0.001) of dyspnea and chest pain. The prevalence of long-COVID beyond 12 weeks was higher in cases than controls (42% vs. 12%; *p* < 0.001) with chest pain (*p* < 0.001) and ageusia (*p* < 0.05), forming the most representative symptoms. **Conclusions**: Symptoms of long-COVID are more frequent in women with than without endometriosis/adenomyosis.

## 1. Introduction

Endometriosis is a chronic gynecological condition of premenopausal women, with about 10% global prevalence and a diagnostic delay ranging from 8 to 10 years [1,2]. It is histologically characterized by ectopic endometrial tissue, typically within the pelvis [3,4], while adenomyosis is characterized by the presence of ectopic endometrial glands and stroma within the myometrium [5]. Menstrual pain, intermenstrual pain, and pain at intercourse are common symptoms associated with these pathologies [4,5], which are treated either by symptomatic medicines or hormones [6,7]. Symptoms and their aggravation are the consequence of a chronic activation of the immune system and marked local inflammation [8,9,10,11,12].

Intense immune activation with a cytokine storm leading to major respiratory and cardiovascular disease were identified as main causes of morbidity and mortality due to SARS-CoV-2 infection. The persistence of immune activation and inflammation induces the persistence of some COVID-19 symptoms, like shortness of breath, chest pain, fatigue, anosmia, ageusia, muscle aches, stress, anxiety, and depression [13,14,15].

In the four years following the COVID-19 pandemic, it has been determined that in one to five affected individuals, symptoms of COVID-19 persist for more than four weeks and in one to ten for more than twelve weeks [13]. The National Institute for Health and Care Excellence (NICE) guidelines define the post-COVID-19 syndrome, or long-COVID, as the persistence of symptoms for more than 12 weeks [14,15]. In spite of immune dysregulation [16,17], contributors to the risk of long-COVID seems to be age and comorbidities like hypertension, dyslipidemia, cardiovascular disease, and endocrine and metabolic dysregulation [17]. 

Symptoms of endometriosis/adenomyosis are increased by SARS-CoV-2 vaccines [18] and by COVID-19 [19]. In addition, a high percentage of women with endometriosis experienced symptoms of post-traumatic stress syndrome after viral infection [20].

The aim of this study is to assess whether the prevalence of long-COVID is different between women with and without endometriosis/adenomyosis. The secondary objective is to evaluate whether the manifestation of long-COVID is different between women with and without endometriosis/adenomyosis.

## 2. Materials and Methods

This study adhered to the Strengthening the Reporting of Observational Studies in Epidemiology (STROBE) guideline [21]. The local ethics committee approved (Comitato Etico Regione Liguria N 260/2022) this observational retrospective study, which was conducted between October 2022 and September 2023 at the outpatient facility for gynecological ultrasonography and at the outpatient facility for endometriosis and chronic pelvic pain at a university hospital. Cases we considered were all consecutive women of reproductive age (18–45 years) with an ultrasound and clinical diagnosis of endometriosis or adenomyosis. Cases were all in regular follow-up and all with a diagnosis before the onset of SARS-CoV-2 infection. Controls we considered were all consecutive women of reproductive age (18–45 years of age) with negative ultrasound findings and the absence of a clinical manifestation of endometriosis or adenomyosis. All controls accessed the ultrasound outpatient facility after SARS-CoV-2 infection. All women affected by SARS-CoV-2 infection (cases or controls) had experienced the infection at least 6 months prior to the actual investigation. SARS-CoV-2 infection had been documented by a positive swab test as reported by the patients. For both groups, the exclusion criteria were women not of reproductive age, pregnancy, and women with autoimmune and chronic inflammatory disease.

### 2.1. Data Collection

At the time of the investigation, baseline information was collected encompassing age, parity, and current hormonal therapies. The revised scoring system of the American Society for Reproductive Medicine (rASRM) was used to stage the cases [22]. The history of SARS-CoV-2 infection was retrospectively evaluated in terms of length of infection, number of infective episodes, total number of vaccine doses received, and symptoms of SARS-CoV-2 infection, like asthenia (defined as weakness or lack of energy in carrying out daily activities), chest pain, dyspnea (defined as shortness of breath), loss of taste (ageusia), and loss of smell (anosmia) [14,23,24]. A secondary questionnaire investigated the long-term persistence of symptoms for a period of over 12 weeks following the initial COVID-19 manifestation [15,25].

### 2.2. Diagnosis of Adenomyosis and Endometriosis

Ultrasound investigations were conducted by level III experienced sonographers utilizing a GE E6 ultrasound machine (GE Medical Systems, Zipf, Austria) and a transvaginal wideband transducer with a frequency range of 5–9 MHz [26].

According to the current literature, transvaginal ultrasound is recommended for the diagnosis of endometriosis or adenomyosis [6,27].

The diagnosis of endometriosis and adenomyosis was conducted using the criteria established by the International Deep Endometriosis Analysis Group (IDEA) and the revised Morphological Uterus Sonographic Assessment, respectively [28,29,30,31]. The International Ovarian Tumor Analysis Consensus (IOTA) was employed for the eventual description of the ultrasound appearance of ovarian masses [32,33,34].

### 2.3. Sample Size

To determine a clinically significant effect size (Cohen’s h) of 0.5, with 90% power at a significance level of 0.05, 76 COVID-19 individuals in the case group and a minimum of 95 COVID-19 individuals in the control group were necessary. The sample size was calculated using a two-proportion different sample size formula where cases were a constant based on women in regular follow-up and controls were variable. Hence, based on the preliminary prevalence data from our setting, we estimated that at least 98 cases and 149 controls would yield the necessary number of women with COVID-19 to guarantee sufficient statistical power for our analysis.

### 2.4. Statistical Analysis

Continuous data’s normality distribution was assessed using the Kolmogorov–Smirnov test. Continuous variable comparisons were conducted using either Student’s t-test or the Wilcoxon rank-sum test, depending on the appropriateness of the data. Categorical variables were analyzed using the chi-square test or Fisher’s exact test, as appropriate. Continuous data were expressed as the mean ± standard deviation (SD) or median with interquartile range (IQR), contingent upon the distribution of the data. Logistic regression analysis was conducted to determine factors linked to the incidence of long-COVID. The dependent variable was the status of long-COVID, indicated by its presence or absence. Independent variables included group classification (cases vs. controls), age, and other possible confounders. Initially, univariate logistic regression was performed for each variable, and those with *p*-values less than 0.1 were incorporated into the multivariate model to account for potential confounders. Results are presented as odds ratios (ORs) and 95% confidence intervals (95%CI). Statistical analyses were performed utilizing R software (version 4.4.2). A *p*-value below 0.05 was deemed statistically significant.

## 3. Results

### 3.1. Population Description

This study included 247 women, of whom 194 had a positive history of SARS-CoV-2 infection and 181 had developed COVID-19 symptoms. None had experience symptoms requiring hospitalization. Among the 247 women, 149 were without (controls) and 98 were with endometriosis or adenomyosis (cases). Endometriosis and adenomyosis were present in 38.8% (38/98) of cases, with endometriosis alone in 36.7% (36/98) and adenomyosis alone in 24.5% (24/98). The median age of women was 27 years (IQR 25–32 years), with controls being significantly younger than cases (27.0 years, IQR 24.0–28.0 years vs. 32.0 years, IQR 27.0–38.7 years, *p* < 0.001) (Table 1). The median BMI of the population was 21.8 (IQR 17–32), differing between cases (22.0; IQR 21.0–24.0) and controls (21.0; IQR 20.0–22.0) (*p* < 0.001) (Table 1). Smokers were similarly distributed between the two groups (Table 1).

Among cases, the median time from the diagnosis of endometriosis or adenomyosis was 38 months (IQR 1–300 months). The median rASRM score was 7 (IQR 2–20), and in 64% of cases, the stage was I or II. Furthermore, there were no significant differences in endometriosis stage between women with (stage II–IV 30%) and without (stage II–IV 35%) long-COVID (*p* = 0.640). The use of hormonal contraceptives or hormonal therapies was more frequent in cases than controls (78.0% vs. 45.6%; *p* < 0.001). The rates of SARS-CoV-2 infection were similar in cases and controls (79.6% vs. 77.8%; *p* < 0.745). The median length of time since SARS-CoV-2 infection was 20 months (IQR 16–23 months), and it was similar in the two groups. The coverage of SARS-CoV-2 vaccination was very high and comparable between the two groups (Table 1).

### 3.2. Symptoms Due to SARS-CoV-2 Infection

Prevalence of COVID-19 symptoms was not significantly different between cases and controls (97.4% vs. 90.5%; *p* = 0.059), but symptoms like dyspnea and chest pain were more frequent (*p* < 0.001) and asthenia less frequent (*p* < 0.001) in cases than in controls (Table 2).

**Figure 1:**
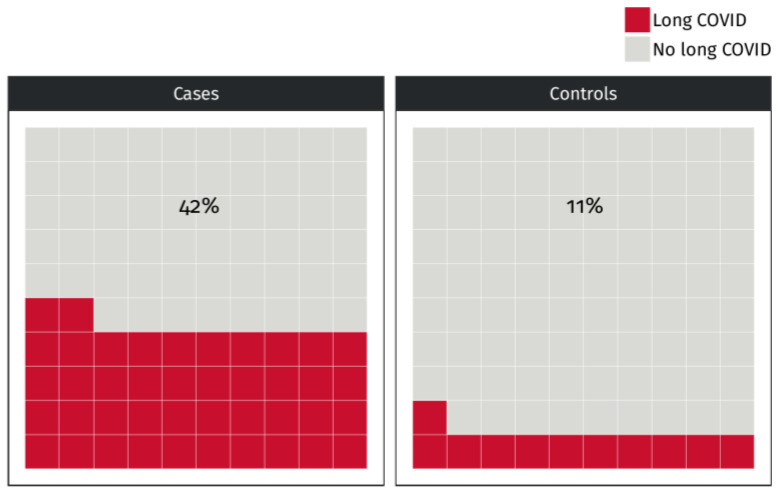
Prevalence of long-COVID in women without (controls) and with (cases) endometriosis/adenomyosis (*p* < 0.001). The *p*-value refers to the chi-square test.

**Figure 2:**
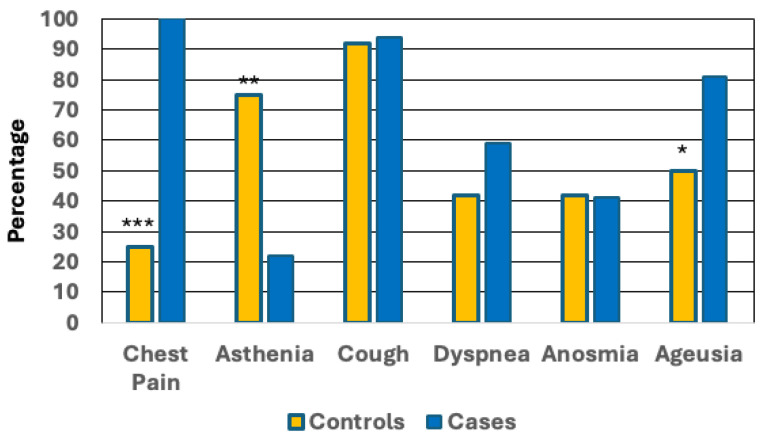
Prevalence of symptoms of long-COVID in women without (controls) and with (cases) endometriosis/adenomyosis with long-COVID. * *p* < 0.05; ** *p* < 0.002; *** *p* < 0.001 vs. cases (the *p*-values refer to the chi-squared test or Fisher’s exact test).

Prevalence of long-COVID was significantly higher in women with than without endometriosis/adenomyosis (42.1% vs. 11.4%; *p* < 0.001) (Table 2, Figure 1). We conducted a sensitivity analysis to evaluate the prevalence of long-COVID across specific subgroups: combined endometriosis and adenomyosis (51.72%, 15/29), isolated endometriosis (40.00%, 12/30), and isolated adenomyosis (29.41%, 5/17). The prevalence in all subgroup cases was significantly higher than that in controls (*p* < 0.05). This difference was still significant after multivariate adjustment for possible contributing factors (Table 3, Model 1). Indeed, the OR remained highly significant when the analysis was adjusted for confounding 5.82 (2.21–15.35, <0.001) (Table 3, Model 1). In the univariate logistic regression analysis, both women’s BMI (OR 1.15, CI.95 1.01–1.31, *p* = 0.032) and age (OR 1.06, CI.95 1.01–1.11, *p* = 0.025) demonstrated significant associations with long COVID. Neither BMI (OR 1.09, CI.95 0.95–1.26, *p* = 0.226) nor age (OR 1.01, CI.95 0.96–1.06, *p* = 0.761) was significant in the multivariate model. The rASRM score demonstrated no significance in both univariate (OR 1.03, CI.95 1.00–1.06, *p* = 0.086) and multivariate analyses (OR 1.00, CI.95 0.96–1.03, *p* = 0.809). A sensitivity analysis revealed significant associations between endometriosis (OR 5.21, CI.95 2.53–10.75, *p* < 0.001) and adenomyosis (OR 3.56, CI.95 1.71–7.39, *p* < 0.001) with long-COVID. However, adenomyosis alone loses significance after adjusting in multivariate models (Table 3, Models 2 and 3). Symptoms of long-COVID differed between cases and controls. Chest pain (*p* < 0.001) and ageusia (*p* < 0.05) were more common, and asthenia (*p* < 0.002) was less common in cases than in controls (Table 2, Figure 2). Use of hormonal therapy or hormonal contraceptives was more frequent in cases than controls (78.0% vs. 45.6%; *p* < 0.001), but it was related neither to the risk of long-COVID nor to its clinical manifestation.

## 4. Discussion

### 4.1. Principal Findings

No significant difference for SARS-CoV-2 infection or vaccination was noted between cases and controls. Prevalence of long-COVID was significantly higher in women with than without endometriosis/adenomyosis, and its clinical manifestation was also different.

### 4.2. Interpretations

There was no significant difference in the prevalence of SARS-CoV-2 infection between women with and without endometriosis or adenomyosis, in accordance with a recent case–control study and meta-analysis [35,36].

The prevalence of long-COVID in controls was like that previously reported in the general population [13] Conversely, in women with endometriosis/adenomyosis, long-COVID was about four times more frequent. Three previous studies have indicated a higher prevalence of long-COVID in women with endometriosis, without evaluating the possible presence of adenomyosis. A two-times-higher risk of long-COVID was reported in women with menstrual pain or endometriosis [37], and a 20% to 43% increased risk in another two studies performed in women with a historical [38] or surgical [39] diagnosis of endometriosis [39]. Immune dysregulation, cytokine storms, gut microbiota alteration, and microvascular complications are likely involved in the pathogenesis of long-COVID [24,40], and immune dysregulation [1,2,11,12], microbiota alteration [41], and micro-clots [42] are commonly found in women with endometriosis/adenomyosis.

Analysis of symptom distribution during acute COVID-19 revealed different patterns between controls, who more frequently suffered from asthenia, and cases, who more frequently suffered from chest pain and dyspnea. Similarly, symptoms of long-COVID were different between cases and controls, with the latter reporting more asthenia, and the former reporting more chest pain with the addition of a more frequent persistence of loss of taste (ageusia). The data align with a previous report showing that women with endometriosis symptoms are different from the general population and often atypical [35]. Hormones may influence SARS-CoV-2 infection and its clinical manifestation, with women of reproductive age behaving differently from women of older age [36,43]. Yet, in women of reproductive age, the use of a hormonal contraceptive had little influence on the manifestation of the COVID-19 syndrome [44]. The present data complement this observation by showing that the use of hormones or hormonal contraceptives does not modify the prevalence or the clinical manifestation of long-COVID.

Similarly to Wang et al., in our multivariate analysis, we found endometriosis to be associated with long-COVID independently of women’s ages and BMIs [39]. Long-COVID was found to be associated with inflammatory clusters [45,46]. Endometriosis is also known to be associated with the activation of the immune system [47,48]. The immune dysregulation linked to endometriosis may result in an increased inflammatory response, potentially complicating the clinical presentation for individuals infected with COVID-19 [23]. This immune response resembles that seen in long-COVID, characterized by persistent inflammation and immune dysregulation [45,46,49]. Also, BMI and age were found to be associated with inflammatory clusters [45], The loss of significance in our multivariate analysis of BMI and women’s age may be related to a smaller effect size of these two factors compared to endometriosis, which can be secondary to a smaller association with inflammatory profiles than endometriosis.

There are only limited data in the literature about the endometriosis rASRM stage and COVID-19, and the results show no significant effect of the endometriosis stage on COVID-19 [23,35]. We confirm the previous literature results, showing no significant impact of the endometriosis stage; in fact, immune dysregulation with a considerable role of macrophages is present in both early and advanced endometriosis stages [23,35].

### 4.3. Strengths and Weaknesses

This study was specifically focused on a population of women with an actual diagnosis of endometriosis/adenomyosis versus a population in which these disturbances were accurately excluded by ultrasonography and clinical symptoms. This is at variance with previous studies based on a past diagnosis of endometriosis, where the actual presence of the disease was confirmed in cases, but not appropriately excluded in controls [37,38,39]. The diagnosis of both adenomyosis and endometriosis was based on ultrasonography and clinical symptoms. For both endometriosis and adenomyosis, as a highly reliable tool to reach an accurate diagnosis of the disease, without the need of surgical procedures [5,29,50].

According to the existing literature, TVS’s specificity for detecting adenomyosis was 75% (CI.95 63–84%), while its sensitivity was 81% (CI.95 60–92%) [51]. Transvaginal ultrasonography has a sensitivity of at least 95% and specificity of at least 50% for endometriomas [52]. The specificity of transvaginal ultrasonography in detecting deep endometriosis varies depending on the site: with the identification of DIE in the rectosigmoid as the most common site, pooled sensitivity was 85% (CI.95 68–94%) and specificity was 96% (CI.95 85–99%) [53].

For endometriosis, a sensitivity of 100% and specificity of 95% were recently reported by our group [54]. If a woman with endometriosis/adenomyosis is assigned as a control or vice versa, this may result in a reduction in the difference in long-COVID prevalence between cases and controls. Accordingly, the main result of this study would not be altered.

Endometriosis and adenomyosis are pathologies that may not coexist [54,55]. This study indicates that adenomyosis may have a comparable effect to endometriosis in affecting the risk of long-COVID. However, in our multivariate models, the association for adenomyosis became non-significant, likely due to the limited number of cases in this subgroup. Although the association’s direction aligned with that of endometriosis, the effect size was diminished, preventing with our data a definitive conclusions about adenomyosis’s isolated impact on long-COVID risk. Moreover, women with both endometriosis and adenomyosis exhibit the highest risk, suggesting that the simultaneous occurrence of these conditions may enhance the overall risk of long-COVID.

Most of the findings we found are consistent with the current literature on long-COVID and inflammatory diseases, and they contribute to increasing the evidence that chronic inflammatory conditions are associated with a heightened risk of long-COVID [1,10,23].

Although adequate for the initial analysis, the sample size may only partially represent the variability present within and between groups, and a larger multicenter study would improve the robustness and reliability of the findings.

A further limitation is the dependence on self-reported symptoms of COVID-19 and long-COVID, potentially leading to recall bias. The comparable time since SARS-CoV-2 infection in both groups reduces the potential influence of recall bias, as both cohorts likely possess a similar capacity to recall and report symptoms.

We did not investigate all the symptoms possibly related to long-COVID. This may have led to underestimation of the rate of long-COVID. Yet, the rate we obtained in the controls was like the one observed in a previous epidemiological study performed in the general population [13], and the rate obtained in the cases was like the one observed in another epidemiological investigation on women with a surgical diagnosis of endometriosis [39].

### 4.4. Generalizability, Clinical Implications, and Research Implications

The generalizability of this study’s findings is affected by specific limitations. The single-center design, concentrated on a specific region and country, may not adequately address variations in healthcare access, diagnostic practices, or population demographics in other regions or countries. Cultural and genetic differences among populations may affect the prevalence and manifestation of both endometriosis/adenomyosis and long-COVID, indicating a need for further studies in diverse contexts. Yet, the similarities between the findings of this study and those in the existing literature enhance its relevance to broader female populations globally, despite the constraints encountered. The persistent patterns of elevated long-COVID prevalence in individuals with inflammatory conditions and the symptom-specific variations noted between cases and controls underscore universal mechanisms that may surpass regional disparities. Future research must address the limitations of this study by incorporating larger, multicenter, international cohorts to enhance the generalizability of the findings and provide a more comprehensive understanding of the intersection between chronic inflammatory diseases and long-COVID. Examining the biological mechanisms that connect endometriosis, adenomyosis, and long-COVID, including immune dysregulation, will provide valuable insights into the identified associations and aid in formulating targeted interventions.

## 5. Conclusions

This study indicates that women with endometriosis or adenomyosis exhibit a markedly higher prevalence of long-COVID in comparison to women without the diseases, even though the rates of initial SARS-CoV-2 infection and vaccination coverage were comparable. These findings indicate that women with endometriosis or adenomyosis are exposed to increased clinical sequelae as the consequence of their diseases, leading to intense and prolonged immune activation.

## Figures and Tables

**Table 1:** Demographic and clinical characteristics in women without (controls) and with (cases) endometriosis or adenomyosis. The *p*-values refer to the chi-squared test and Wilcoxon test.

Variables	Controls (149)	Cases (98)	*p*
Patient characteristics			
Women age (years)	27.0 (24.0–28.0) (*)	31.0 (27.0–38.7) (*)	<0.001
Nulliparity	0%	20.6% (†: 1)	<0.001
BMI	21.0 (20.0–22.0) (*)	22.0 (21.0–24.0) (*)	<0.001
Smoking	20.8%	18.4%	0.638
Hormonal therapy/contraception	45.6%	78.0% (†: 7)	<0.001
SARS-CoV-2 vaccine	98.7%	95.9% (†: 1)	0.167
SARS-CoV-2 vaccine doses			0.456
One dose	3.4% (†: 2)	5.4% (†: 5)	
Two/three doses	96.6% (†: 2)	94.6% (†: 5)	
SARS-CoV-2 infection	77.8%	79.6%	0.745
Time since SARS-CoV-2 infection (months)	20.0 (16.0–23.0) (*)	20.0 (17.0–23.0) (*)	0.918

(*) median and interquartile range (IQR). (†) number of missing values.

**Table 2:** Analysis of COVID-19 symptom distribution and long-COVID prevalence in women without (controls) and with endometriosis or adenomyosis (cases). The *p*-values refer to the chi-squared test or Fisher’s exact test.

Variables	Controls	Cases	*p*
Number	116	78	
Symptomatic COVID-19	90.5%	97.4%	0.059
Number	105	76	
COVID19 symptoms distribution			
Chest pain	12.4%	96.0%	<0.001
Asthenia	68.6%	14.5%	<0.001
Cough	78.1%	88.2%	0.080
Dyspnea	26.7%	67.1%	<0.001
Anosmia	30.5%	36.8%	0.369
Ageusia	34.3%	38.2%	0.592
Number	105	76	
Frequency of Long-COVID	11.43%	42.1%	<0.001
Number	12	32	
COVID19 symptoms distribution in long COVID			
Chest pain	25.0%	100.0%	<0.001
Asthenia	75.0%	21.9%	<0.001
Cough	91.7%	93.7%	0.807
Dyspnea	41.7%	59.4%	0.293
Anosmia	41.7%	40.6%	0.950
Ageusia	50.0%	81.2%	0.038

**Table 3:** Simple and multiple (*) logistic regression analyses of factors associated with long COVID. This table displays the main analysis (Model 1) and two sensitivity analyses (Models 2 and 3). In Model 2, endometriosis and adenomyosis are analyzed as dummy variables, while in Model 3 the three subgroups (endometriosis and adenomyosis combined, endometriosis only, and adenomyosis only) are examined separately.

Variables	N	OR (CI.95)	*p*	OR (CI.95) (*)	*p* (*)
**Main analysis**					
*Long COVID*		*Model 1*		*Model 1 (†)*	
Groups					
Controls	105	Reference	1.000	Reference	1.000
Cases (endometriosis/adenomyosis)	76	5.64 (2.65–11.98)	0.001	5.82 (2.21–15.35)	<0.001
**Sensitivity analyses**					
*Long COVID*		*Model 2*		*Model 2 (†)*	
Endometriosis	59	5.21 (2.53–10.75)	<0.001	5.16 (1.98–13.39)	<0.001
Adenomyosis	46	3.56 (1.71–7.39)	<0.001	2.39 (0.99–5.82)	0.054
*Long COVID*		*Model 3*		*Model 3 (‡)*	
Groups					
Controls	105	Reference	1.000	Reference	1.000
Endometriosis and adenomyosis	29	7.99 (3.12–20.43)	<0.001	6.80 (2.52–18.36)	<0.001
Only endometriosis	30	5.05 (1.97–12.93)	<0.001	4.44 (1.68–11.77)	0.003
Only adenomyosis	17	3.30 (1.01–10.85)	0.049	2.73 (0.78–9.54)	0.117

Controls = Women without endometriosis or adenomyosis; Cases = Women with endometriosis or adenomyosis. OR = Odds Ratio; CI.95 = 95% Confidence Interval. (*) Multivariate models. (†) Multivariate model adjusted for age, BMI, hormonal therapy/contraception, and rASRM score (included if *p* < 0.100 in univariate analysis). (‡) Multivariate model adjusted for age and BMI; variables were included only if *p* < 0.05 in the univariate analysis, given the smaller sample sizes.

## Data Availability

The data that support the findings of this study are available. However, restrictions apply to the availability of these data, which were used under license for the current study and are not publicly available. Data are, however, available from the authors upon reasonable request and with permission of the local ethics committee.

## References

[1] Lamceva J., Uljanovs R., Strumfa I. (2023). The Main Theories on the Pathogenesis of Endometriosis. Int. J. Mol. Sci..

[2] Ahn S.H., Singh V., Tayade C. (2017). Biomarkers in Endometriosis: Challenges and Opportunities. Fertil. Steril..

[3] Camboni A., Marbaix E. (2021). Ectopic Endometrium: The Pathologist’s Perspective. Int. J. Mol. Sci..

[4] Saunders P.T.K., Horne A.W. (2021). Endometriosis: Etiology, Pathobiology, and Therapeutic Prospects. Cell.

[5] Bourdon M., Santulli P., Marcellin L., Maignien C., Maitrot-Mantelet L., Bordonne C., Plu Bureau G., Chapron C. (2021). Adenomyosis: An Update Regarding Its Diagnosis and Clinical Features. J. Gynecol. Obstet. Hum. Reprod..

[6] Becker C.M., Bokor A., Heikinheimo O., Horne A., Jansen F., Kiesel L., King K., Kvaskoff M., Nap A., Petersen K. (2022). ESHRE Guideline: Endometriosis. Hum. Reprod. Open.

[7] Etrusco A., Barra F., Chiantera V., Ferrero S., Bogliolo S., Evangelisti G., Oral E., Pastore M., Izzotti A., Venezia R. (2023). Current Medical Therapy for Adenomyosis: From Bench to Bedside. Drugs.

[8] Sampson J.A. (1940). The Development of the Implantation Theory for the Origin of Peritoneal Endometriosis. Am. J. Obstet. Gynecol..

[9] Yovich J.L., Rowlands P.K., Lingham S., Sillender M., Srinivasan S. (2020). Pathogenesis of Endometriosis: Look No Further than John Sampson. Reprod. Biomed. Online.

[10] Sampson J.A. (1927). Metastatic or Embolic Endometriosis, Due to the Menstrual Dissemination of Endometrial Tissue into the Venous Circulation. Am. J. Pathol..

[11] Abramiuk M., Grywalska E., Małkowska P., Sierawska O., Hrynkiewicz R., Niedźwiedzka-Rystwej P. (2022). The Role of the Immune System in the Development of Endometriosis. Cells.

[12] Zhai J., Vannuccini S., Petraglia F., Giudice L.C. (2020). Adenomyosis: Mechanisms and Pathogenesis. Semin. Reprod. Med..

[13] Lechner-Scott J., Levy M., Hawkes C., Yeh A., Giovannoni G. (2021). Long COVID or Post COVID-19 Syndrome. Mult. Scler. Relat. Disord..

[14] Carfì A., Bernabei R., Landi F. (2020). Gemelli Against COVID-19 Post-Acute Care Study Group Persistent Symptoms in Patients After Acute COVID-19. JAMA.

[15] Soriano J.B., Murthy S., Marshall J.C., Relan P., Diaz J.V. (2022). A Clinical Case Definition of Post-COVID-19 Condition by a Delphi Consensus. Lancet Infect. Dis..

[16] Pretorius E., Venter C., Laubscher G.J., Kotze M.J., Oladejo S.O., Watson L.R., Rajaratnam K., Watson B.W., Kell D.B. (2022). Prevalence of Symptoms, Comorbidities, Fibrin Amyloid Microclots and Platelet Pathology in Individuals with Long COVID/Post-Acute Sequelae of COVID-19 (PASC). Cardiovasc. Diabetol..

[17] Tenforde M.W., Kim S.S., Lindsell C.J., Billig Rose E., Shapiro N.I., Files D.C., Gibbs K.W., Erickson H.L., Steingrub J.S., Smithline H.A. (2020). Symptom Duration and Risk Factors for Delayed Return to Usual Health Among Outpatients with COVID-19 in a Multistate Health Care Systems Network—United States, March-June 2020. MMWR Morb. Mortal. Wkly. Rep..

[18] Xholli A., Schiaffino M.G., Vacca I., Molinari F., Cavalli E., Scovazzi U., Oppedisano F., Jakimovska M., Londero A.P., Cagnacci A. (2023). Effect of COVID-19 Vaccine in Women with Adenomyosis and Endometriosis. Clin. Exp. Obstet. Gynecol..

[19] Kabani Z., Ramos-Nino M.E., Ramdass P.V.A.K. (2022). Endometriosis and COVID-19: A Systematic Review and Meta-Analysis. Int. J. Mol. Sci..

[20] Arena A., Orsini B., Degli Esposti E., Raimondo D., Lenzi J., Verrelli L., Iodice R., Casadio P., Seracchioli R. (2021). Effects of the SARS-CoV-2 Pandemic on Women Affected by Endometriosis: A Large Cross-Sectional Online Survey. Ann. Med..

[21] von Elm E., Altman D.G., Egger M., Pocock S.J., Gøtzsche P.C., Vandenbroucke J.P., STROBE Initiative (2007). The Strengthening the Reporting of Observational Studies in Epidemiology (STROBE) Statement: Guidelines for Reporting Observational Studies. Ann. Intern. Med..

[22] American Society For Reproductive Medicine (1997). Revised American Society for Reproductive Medicine Classification of Endometriosis: 1996. Fertil. Steril..

[23] Barretta M., Savasta F., Pietropaolo G., Barbasetti A., Barbera V., Vignali M. (2022). COVID-19 Susceptibility in Endometriosis Patients: A Case Control Study. Am. J. Rep. Immunol..

[24] Davis H.E., McCorkell L., Vogel J.M., Topol E.J. (2023). Long COVID: Major Findings, Mechanisms and Recommendations. Nat. Rev. Microbiol..

[25] Whitaker M., Elliott J., Chadeau-Hyam M., Riley S., Darzi A., Cooke G., Ward H., Elliott P. (2022). Persistent COVID-19 Symptoms in a Community Study of 606,434 People in England. Nat. Commun..

[26] (2006). European Federation of Societies for Ultrasound in Medicine and Biology. Ultraschall Med..

[27] Condous G., Gerges B., Thomassin-Naggara I., Becker C., Tomassetti C., Krentel H., Van Herendael B.J., Malzoni M., Abrao M.S., Saridogan E. (2024). Non-invasive Imaging Techniques for Diagnosis of Pelvic Deep Endometriosis and Endometriosis Classification Systems: An International Consensus Statement. Ultrasound Obstet. Gynecol..

[28] Guerriero S., Condous G., Van Den Bosch T., Valentin L., Leone F.P.G., Van Schoubroeck D., Exacoustos C., Installé A.J.F., Martins W.P., Abrao M.S. (2016). Systematic Approach to Sonographic Evaluation of the Pelvis in Women with Suspected Endometriosis, Including Terms, Definitions and Measurements: A Consensus Opinion from the International Deep Endometriosis Analysis (IDEA) Group. Ultrasound Obstet. Gynecol..

[29] Van Den Bosch T., Dueholm M., Leone F.P.G., Valentin L., Rasmussen C.K., Votino A., Van Schoubroeck D., Landolfo C., Installé A.J.F., Guerriero S. (2015). Terms, Definitions and Measurements to Describe Sonographic Features of Myometrium and Uterine Masses: A Consensus Opinion from the Morphological Uterus Sonographic Assessment (MUSA) Group. Ultrasound Obstet. Gynecol..

[30] Harmsen M.J., Van den Bosch T., de Leeuw R.A., Dueholm M., Exacoustos C., Valentin L., Hehenkamp W.J.K., Groenman F., De Bruyn C., Rasmussen C. (2022). Consensus on Revised Definitions of Morphological Uterus Sonographic Assessment (MUSA) Features of Adenomyosis: Results of Modified Delphi Procedure. Ultrasound Obstet. Gynecol..

[31] Pedrassani M., Guerriero S., Pascual M.Á., Ajossa S., Graupera B., Pagliuca M., Podgaec S., Camargos E., Vieira de Oliveira Y., Alcázar J.L. (2023). Superficial Endometriosis at Ultrasound Examination—A Diagnostic Criteria Proposal. Diagnostics.

[32] Epstein E., Fischerova D., Valentin L., Testa A.C., Franchi D., Sladkevicius P., Frühauf F., Lindqvist P.G., Mascilini F., Fruscio R. (2018). Ultrasound Characteristics of Endometrial Cancer as Defined by International Endometrial Tumor Analysis (IETA) Consensus Nomenclature: Prospective Multicenter Study: Ultrasound Characteristics of Endometrial Cancer. Ultrasound Obstet. Gynecol..

[33] Timmerman D., Valentin L., Bourne T.H., Collins W.P., Verrelst H., Vergote I. (2000). Terms Definitions and Measurements to Describe the Sonographic Features of Adnexal Tumors: A Consensus Opinion from the International Ovarian Tumor Analysis (IOTA) Group. Ultrasound Obstet. Gynecol..

[34] Van Holsbeke C., Van Calster B., Guerriero S., Savelli L., Paladini D., Lissoni A.A., Czekierdowski A., Fischerova D., Zhang J., Mestdagh G. (2010). Endometriomas: Their Ultrasound Characteristics. Ultrasound Obstet. Gyne.

[35] Moazzami B., Chaichian S., Samie S., Zolbin M.M., Jesmi F., Akhlaghdoust M., Pishkuhi M.A., Mirshafiei Z.S., Khalilzadeh F., Safari D. (2021). Does Endometriosis Increase Susceptibility to COVID-19 Infections? A Case-Control Study in Women of Reproductive Age. BMC Women’s Health.

[36] Riemma G., Etrusco A., Laganà A.S., Torella M., Vastarella M.G., Della Corte L., D’Amato A., La Verde M., De Franciscis P., Cobellis L. (2024). Susceptibility to Infection and Impact of COVID-19 Vaccines on Symptoms of Women with Endometriosis: A Systematic Review and Meta-Analysis of Available Evidence. Reprod. Sci..

[37] Cirkel A., Göbel H., Göbel C., Alkatout I., Khalil A., Baum S., Brüggemann N., Rody A., Cirkel C. (2024). Endometriosis Patients Have an Increased Risk of Experiencing Long-Covid Symptoms: Results from a Cross-Sectional Multicenter Study. Women’s Health Rep..

[38] Subramanian A., Nirantharakumar K., Hughes S., Myles P., Williams T., Gokhale K.M., Taverner T., Chandan J.S., Brown K., Simms-Williams N. (2022). Symptoms and Risk Factors for Long COVID in Non-Hospitalized Adults. Nat. Med..

[39] Wang S., Farland L.V., Gaskins A.J., Mortazavi J., Wang Y.-X., Tamimi R.M., Rich-Edwards J.W., Zhang D., Terry K.L., Chavarro J.E. (2023). Association of Laparoscopically-Confirmed Endometriosis with Long COVID-19: A Prospective Cohort Study. Am. J. Obstet. Gynecol..

[40] Kell D.B., Laubscher G.J., Pretorius E. (2022). A Central Role for Amyloid Fibrin Microclots in Long COVID/PASC: Origins and Therapeutic Implications. Biochem. J..

[41] Xholli A., Cremonini F., Perugi I., Londero A.P., Cagnacci A. (2023). Gut Microbiota and Endometriosis: Exploring the Relationship and Therapeutic Implications. Pharmaceuticals.

[42] Li Y., Liu H., Ye S., Zhang B., Li X., Yuan J., Du Y., Wang J., Yang Y. (2023). The Effects of Coagulation Factors on the Risk of Endometriosis: A Mendelian Randomization Study. BMC Med..

[43] Cagnacci A., Xholli A. (2021). Change in COVID-19 Infection and Mortality Rates in Postmenopausal Women. Menopause.

[44] Harrington L.B., Powers J.D., Bayliss E.A., Fortmann S.P., Shortreed S.M., Walker R.L., Floyd J.S., Kuntz J., Fuller S., Alberston-Junkans L. (2024). Current Use of Estrogen-Containing Oral Contraceptives or Hormone Therapy and Risk of COVID-19 Infection and Hospitalization: A Population-Based Cohort Study. Am. J. Epidemiol..

[45] Dhingra S., Fu J., Cloherty G., Mallon P., Wasse H., Moy J., Landay A., Kenny G. (2024). Identification of Inflammatory Clusters in Long-COVID through Analysis of Plasma Biomarker Levels. Front. Immunol..

[46] Wynberg E., Han A.X., Van Willigen H.D.G., Verveen A., Van Pul L., Maurer I., Van Leeuwen E.M., Van Den Aardweg J.G., De Jong M.D., Nieuwkerk P. (2024). Inflammatory Profiles Are Associated with Long COVID up to 6 Months after COVID-19 Onset: A Prospective Cohort Study of Individuals with Mild to Critical COVID-19. PLoS ONE.

[47] Mariuzzi L., Domenis R., Orsaria M., Marzinotto S., Londero A.P., Bulfoni M., Candotti V., Zanello A., Ballico M., Mimmi M.C. (2016). Functional Expression of Aryl Hydrocarbon Receptor on Mast Cells Populating Human Endometriotic Tissues. Lab. Investig..

[48] Akoum A., Lemay A., Paradis I., Rheault N., Maheux R. (1996). Endometriosis: Secretion of Interleukin-6 by Human Endometriotic Cells and Regulation by Proinflammatory Cytokines and Sex Steroids. Hum. Reprod..

[49] Davis H.E., Assaf G.S., McCorkell L., Wei H., Low R.J., Re’em Y., Redfield S., Austin J.P., Akrami A. (2021). Characterizing Long COVID in an International Cohort: 7 Months of Symptoms and Their Impact. eClinicalMedicine.

[50] Chapron C., Vannuccini S., Santulli P., Abrão M.S., Carmona F., Fraser I.S., Gordts S., Guo S.-W., Just P.-A., Noël J.-C. (2020). Diagnosing Adenomyosis: An Integrated Clinical and Imaging Approach. Hum. Reprod. Update.

[51] Alcázar J.L., Vara J., Usandizaga C., Ajossa S., Pascual M.Á., Guerriero S. (2023). Transvaginal Ultrasound versus Magnetic Resonance Imaging for Diagnosing Adenomyosis: A Systematic Review and Head-to-head Meta-analysis. Int. J. Gynecol. Obstet..

[52] Kanti F.S., Gorak Savard R., Bergeron F., Zomahoun H.T.V., Netter A., Maheux-Lacroix S. (2024). Transvaginal Ultrasound and Magnetic Resonance Imaging in the Diagnosis of Endometrioma: A Systematic Review and Meta-Analysis of Diagnostic Test Accuracy Studies. J. Obstet. Gynaecol..

[53] Guerriero S., Saba L., Pascual M.A., Ajossa S., Rodriguez I., Mais V., Alcazar J.L. (2018). Transvaginal Ultrasound vs Magnetic Resonance Imaging for Diagnosing Deep Infiltrating Endometriosis: Systematic Review and Meta-Analysis. Ultrasound Obstet. Gynecol..

[54] Xholli A., Molinari F., Scovazzi U., Londero A.P., Perugi I., Kratochwila C., Cremonini F., Cagnacci A. (2024). Relationship between Endometriosis and Uterine Cervical Elasticity Assessed Using Ultrasound Strain Elastography. Ultrasonography.

[55] Xholli A., Scovazzi U., Londero A.P., Evangelisti G., Cavalli E., Schiaffino M.G., Vacca I., Oppedisano F., Ferraro M.F., Sirito G. (2022). Angle of Uterine Flexion and Adenomyosis. J. Clin. Med..

